# Green Synthesis of Fe–Cu Bimetallic Supported on Alginate-Limestone Nanocomposite for the Removal of Drugs from Contaminated Water

**DOI:** 10.3390/polym15051221

**Published:** 2023-02-28

**Authors:** Inas A. Ahmed, Hala. S. Hussein, Zeid A. ALOthman, Abdullah G. ALanazi, Norah Salem Alsaiari, Awais Khalid

**Affiliations:** 1 Department of Chemistry, Faculty of Science, King Khalid University, Abha 62224, Saudi Arabia; 2 Chemical Engineering & Pilot Plant Department, Engineering Research and Renewable Energy Institute, National Research Centre, Cairo 11865, Egypt; 3 Chemistry Department, College of Science, King Saud University, Riyadh 11451, Saudi Arabia; 4 Department of Chemistry, College of Science, Princess Nourah bint Abdulrahman University, P.O. Box 84428, Riyadh 11671, Saudi Arabia; 5 Department of Physics, Hazara University, Mansehra 21300, Khyber Pakhtunkhwa, Pakistan

**Keywords:** ciprofloxacin (CIP), levofloxacin (LEV), bimetallic, limestone, alginate, adsorption

## Abstract

In this study Fe–Cu supported on Alginate-limestone (Fe–Cu/Alg–LS) was prepared. The increase in surface area was the main motivation for the synthesis of ternary composites. Scanning electronic microscopy (SEM), energy-dispersive X-ray spectroscopy (EDX), and transmission electron microscopy (TEM) were used to examine the surface morphology, particle size, percentage of crystallinity, and elemental content of the resultant composite. Fe–Cu/Alg–LS was used as an adsorbent for the removal of drugs such as ciprofloxacin (CIP) and levofloxacin (LEV)from contaminated medium. The adsorption parameters were computed using kinetic and isotherm models. The maximum removal efficiency of CIP (20 ppm) and LEV (10 ppm) was found to be 97.3% and 100%, respectively. The optimal conditions were pH 6 and 7 for CIP and LEV, optimum contact time 45, 40 min for CIP and LEV, and temperature of 303 K. The pseudo-second-order model, which confirmed the chemisorption properties of the process, was the most appropriate kinetic model among the ones used, and the Langmuir model, which was the most appropriate isotherm model. Moreover, the parameters of thermodynamics were also assessed. The results imply that the synthesized nanocomposites can be used to remove hazard materials from aqueous solutions.

## 1. Introduction

In the last decade, pharmaceutical residues, typically at low concentrations, have been detected in the water cycle, including surface waters, wastewater, groundwater and, to a lesser extent, drinking-water. Their presence in water, even at very low concentrations is a potential risk not only to human health from exposure to traces of pharmaceuticals via drinking-water, but also to aquatic organisms and other components of the environment [1,2]. Accordingly, there is a significant need to propose cost-effective and feasible alternatives to eliminate these toxic products from the environment. In this investigation, the fluoroquinolone antibiotics ciprofloxacin (CIP) and levofloxacin (LEV) are used as model antibiotics. In river water, CIP and LEV antibiotics were found in amounts ranging from 0.2 to 18.8 ng/L and 0.3 to 6.0 ng/L, whereas industrial wastewater had concentrations of 0.91 to 99.3 ng/L and 0.5 to 19,981 ng/L, respectively [3]. These antibiotics were detected in drinking water after conventional water treatment techniques such as flocculation, sedimentation, and chlorination. As a result, alternative technologies for treating effluents containing antibiotics, such as electrocoagulation [4], membrane filtration [5], advanced oxidative process [6], or adsorption, have received a lot of attention [7,8,9]. The adsorption process has been suggested as a more viable approach than alternative treatment methods due to a number of advantages, including cheap cost, reusability, accessibility and flexibility, ease of operation, and nonsusceptibility to contaminants and dangerous compounds [10]. Recently, there is a tremendous increase in research for using green nanomaterials as an adsorbent, with an emphasis on their possible use in environmental remediation. Metal-based nanoparticles (for instance, nCu or nFe) are one type of nanomaterial that is frequently suggested for wastewater treatment because of their advantages of higher intrinsic reactivity on their surface sites due to their small particle size and large specific surface area, which is frequently suitable for removal of various organic and inorganic contaminants from surface or ground waters [10,11,12,13,14].

Alginate is a naturally occurring polysaccharide that is commercially harvested from brown algae. It has a highly functionalized backbone (mostly hydroxyl and carboxylic groups) and can be used to create materials that are ionically crosslinked with divalent cations. It has additional properties such as biodegradability, renewability, and nontoxicity which encouraged its contribution with other adsorbents to water treatment [15]. Alginate can be combined with other materials, such as chitosan, hydroxyapatite, or activated carbon to create materials with a variety of properties used in several fields, such as medicine, pharmacy, and environmental protection. Hence, Wasilewska and Deryo-Marczewska used alginate–carbon composites as an effective adsorbent for the removal of non-steroidal anti-inflammatory drugs [16]. Moreover, limestone is a common adsorbent due to its reasonable price and widespread availability in nature. It has been shown that limestone can remove various contaminants, including heavy metals, dyes, and pharmaceuticals. In addition to its heterogeneous surface, the buffering capability, secondary binding site, and repurposing properties are particularly helpful. As a result, limestone can be used as an inexpensive adsorbent in the treatment of water [17].

Green synthesis provides several advantages over conventional synthesis technologies, including being more freely diffused and using less chemicals that are naturally harmful and dangerous. For the creation of metal-based nanoparticles, several natural plants, including green tea, have been employed [18]. According to Zhu et al.’s study [19] on the green synthesis of nano zero-valent iron/Cu using green tea, this approach was shown as more affordable and environmentally friendly than traditional ones. Polyphenols, flavonoids, and other reducing agents found in plant extracts can effectively reduce ferric or ferrous ions and prevent the agglomeration of nanoparticles. Hu et al., [20] investigated the removal of ciprofloxacin with aluminum-pillared kaolin sodium alginate beads with maximum adsorption capacity 68.36 mg/g at pH = 4. Moreover, levofloxacin (LEV) was examined by Al-Jabari et al. [21] for its ability to bind to the surface of superparamagnetic iron oxide nanoparticles (Fe_3_O_4_) and Fe_3_O_4_ & SiO_2_ nanocomposites. At pH 6.5 and 240 min contact time, the greatest removal effectiveness of 80.2% of LEV was attained.

In this study, zero valence Fe–Cu nanoparticles supported on alginate-limestone (ZVFe–Cu/Alg–LS) were prepared as a successful nanocomposite material for eliminating antibiotics from water. The nanocomposite (ZVFe–Cu/Alg–LS) is presented as a group of adsorbents (zero valence Fe–Cu nanoparticles, alginate polymer and limestone) exhibting high ability to adsorb and degrade the fluoroquinolone antibiotics. It is an environmentally friendly nanocomposite that includes several active sites synthesized for removal of CIP and LEV antibiotics. The efficient removal of CIP and LEV was predicted to benefit from the synergistic effect of the (ZVFe–Cu/Alg–LS) nanocomposites. The operating conditions such as the solution pH, drug concentration, contact time and temperature were investigated beside Langmuir and Freundlich isotherm models. In addition, the kinetic models, were also applied to analyze the experimental equilibrium data.

## 2. Experimental

### 2.1. Materials

The materials utilized included limestone from Al-Gomhoria Company (Al-Mansoura, Egypt), calcium chloride (CaCl_2_), sodium alginate, potassium dichromate (K_2_Cr_2_O_7_), copper (II) sulphate pentahydrate (CuSO_4_·5H_2_O), and ferrous sulfate (FeSO_4_.7H_2_O) acquired from Sigma-Aldrich. Green tea leaves obtained from a local market (Egypt). Amon Company, Cairo, Egypt, provided the ciprofloxacin (20 ppm) and levofloxacin (10 ppm). Table 1 lists the physicochemical properties of ciprofloxacin and levofloxacin. The sample’s pH was accustomed using sodium hydroxide (NaOH) and hydrochloric acid (HCl). None of the chemicals had been refined and were all of commercial purity.

### 2.2. Synthesis of Copper Nanoparticles

The production of green copper nanoparticles was performed using green tea leaf extracts, according to the technique explained by Asghar [25]. Green tea leaves were purchased from a local market in Cairo (Egypt). The leaf extracts of each tea were separated by exactly weighing 10 g of tea leaves and transferring into a 250 mL conical flask already containing 100 mL of DI-H_2_O. The mixtures were then heated at 80 °C for 10 min, cooled and filtered. Copper nanoparticles from black tea were synthesized using a CuSO_4_ solution with the corresponding tea leaves extract. Briefly, CuSO_4_ (1 mmol/L) and tea leaf extracts were used in a 4:1 ratio by volume, and the solution was subjected to continuous stirring at 80 °C for 10 min. The resultant suspensions were settled at room temperature for 24 h to complete the reaction, then filtered and washed three times with DI-H_2_O to remove any unbound molecules. Lastly, Cu-NPs were dried at 65 °C for 3 h.

### 2.3. Synthesis of Fe^−^ Nanoparticles

Black tea leaves were used to prepare Fe^−^ nanoparticles. Black tea leaves was washed many times with tap water and then with double distilled water to eliminate impurities. The washed tea was dried at 105 °C for 24 h in a hot air oven and then powder form obtained after grinding of dried tea. Extraction procedure was carried out according to Anamika et al. [14]. An extraction of tea sample was prepared with a 1:10 ratio of tea powder to water, and the extraction was carried out at 80 °C for 30 min. Then the extract was filtered after cooling at room temperature and then 0.1 M of FeSO_4_ solution was added to the tea extract at a ratio of 1:3 (1-part FeSO_4_ and 3-part extract) [26,27]. Then sodium hydroxide solution was added, dropwise. During this process, the ferrous ions were converted into nanometallic particles rapidly. Separation of prepared black nanoparticles was performed by filtration and then washed with double distilled water. Separated nanoparticles were subsequently dried in a vacuum at 50 °C for 24 h.

### 2.4. Preparation of ZV Fe–Cu/Alg–LS Nanocomposites

In order to formulate ZV Fe–Cu/Alg–LS nanocomposites, 2% (*w*/*v*) adsorbents of sodium alginate and 7 g of limestone were mixed in 100 mL of distilled water. The combination was stirred with a mechanical stirrer and heated on a hot plate to 80 °C. When the mixture of limestone and alginate attained a homogeneous condition, a solution of mixed zero valance Fe–Cu (0.5 Fe–0.5 Cu in 100 mL) was added. Then 0.3 M of calcium chloride was dripped through a syringe injector into to form beads. In order to obtain hardened beads, the beads were submerged in a calcium chloride (CaCl_2_) solution for 12 h. Washing the adsorbents with distilled water several times removed the excess unbounded calcium chloride from the adsorbent surface.

### 2.5. Surface Characterization of the Nanocomposites

#### 2.5.1. Instruments

The investigation of the adsorbent was carried out using a Genesis-II FTIR spectrometer (ALT, San Diego, CA, USA) (using potassium bromide). Additionally, (SEM) was performed using an Inspect S (FEI Company, Eindhoven, the Netherlands) equipped with an energy-dispersive X-ray analyzer (EDX, Quanta 200, FEL, Eindhoven, the Netherlands).The mineralogical structure of the powdered materials was determined using X-ray diffraction (XRD) and logged on a Philips PW 1050/70 diffractometer (Philips, Am-sterdam, the Netherlands) using a Cu–Kα source with a post-sample Kα filterant, a scanning speed of 1 s/step, a range of 5 to 50 (2θ°) and a resolution of 0.05°/step. The surface area was determined using BEL SORB max (Made in Japan). TEM analysis was performed using the JEM-HR-2001 model (JEOL, Akishima, Japan) with an accelerating voltage of 200 kV to assess the particle sizes of the material. CIP and LEV were detected in aqueous solutions using an Agilent HPLC 1200 Infinity apparatus equipped with a photodiode array detector (Agilent Technologies, Waldbronn, Germany). At 280 nm, the chromatograms were captured. At a temperature of 25 C, an Agilent Zorbax Eclipse Plus C18 column (3.5 mm, 150 mm, 4.6 mm) (Agilent, Newport, CA, USA) was used. A total of 40% water (mobile phase A) and 60% acetonitrile made up (mobile phase C). The flow rate was set at 1.0 mL/min. BEL and SORB max (Made in Japan) assisted in measuring the surface’s area, and an OHAUS STARTER 2100 pH meter (Pine Brook, NJ, USA) was used for pH adjustment [28].

#### 2.5.2. Adsorption Process

The adsorption was carried out by mixing an identified quantity of the sorbent with an aqueous solution of CIP and LEV at the necessary concentration in a 100 mL capped flask using a mechanical stirrer. First, a certain quantity of the sorbent and 25 mL of the sorbate solution were mixed and agitated for long enough to allow for sorption equilibrium. After filtering the mixtures using filter paper, HPLC was used to calculate the concentration of the antibiotic and medication in the solution. By adjusting the contact duration t (10–90 min), pharmaceutical solution starting concentration (10–100 ppm), and drug solution beginning pH (2–10) using 0.1 M NaOH and HCl, researchers were able to study the effects of a variety of parameters on sorption. The proportion of antibiotics sorption was assessed by the incoming Equations (1) and (2) [29].

(1) $$\mathrm{Adsorption}\;\mathrm{capacity}\;q_{e}=\frac{\left(C_{0}-C_{e}\right)V}{W}$$

(2) $$\mathrm{Removal}\;\mathrm{efficiency}\;\%=\frac{\left(C_{0}-C_{e}\right)}{C_{0}}\times 100$$

where V (L) and W (g) stand for the volume of the solution and the weight of the adsorbent, respectively, C_0_ and C_e_ also stand for the initial and equilibrium concentrations (mg/L) of CIP and LEV ions, respectively.

## 3. Results and Discussion

### 3.1. Zero Valant Fe–Cu/Alg–LS Nanocomposites Characteristics

#### 3.1.1. FTIR Study

**Figure 1:**
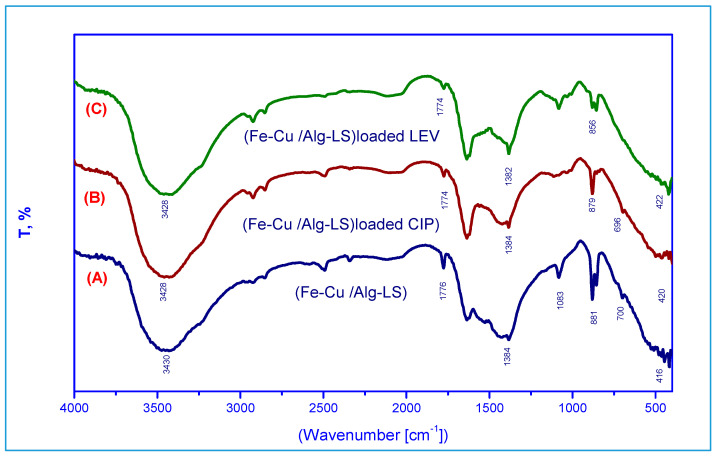
FTIR spectra of (**A**) the Fe–Cu/Alg–LS nanocomposite, (**B**) Fe–Cu/Alg–LS—loaded CIP, (**C**) Fe–Cu/Alg–LS—loaded LEV.

Figure 1 shows the Fe–Cu/Alg–LS nanocomposites’ Fourier-transform infrared spectra before and after CIP and LEV adsorption. A specific band that was associated with O-H (hydroxyl) groups appeared at about 3440 cm^−1^. The peaks observed at 1334 and 1081 cm^−1^ suggest the existence of OH bending and C-O stretching vibrations [30]. At 1774 cm^−1^, two additional peaks can be seen that are associated to the stretching vibration of C=O seen in carboxylic and/or carbonyl moiety groups [31]. Additionally, a peak at 700 cm^−1^ that was correspond to C-H out-of-plane bending in benzene derivatives [32] was observed. As demonstrated in Figure 1B, in Fe–Cu/Alg–LS loaded CIP, After the adsorption, numerous functional groups adjusted to different bands, it was noticed that the bands at 3430, 1776, 881, 700 and 416 cm^−1^ shifted to 3428,1774, 879, 696 and 420 cm^−1^, respectively. Moreover, after adsorption of levofloxacin (LEV) the bands at 3430, 1776, 1384, 881, 700 and 416 cm^−1^ shifted to at 3482, 1774, 1382, 856 and 422 cm^−1^, respectively. Shifting the bands can explain the presence of H-bonded OH in the adsorption of CIP and LEV on Fe–Cu/Alg–LS nanocomposites.

#### 3.1.2. XRD Study

**Figure 2:**
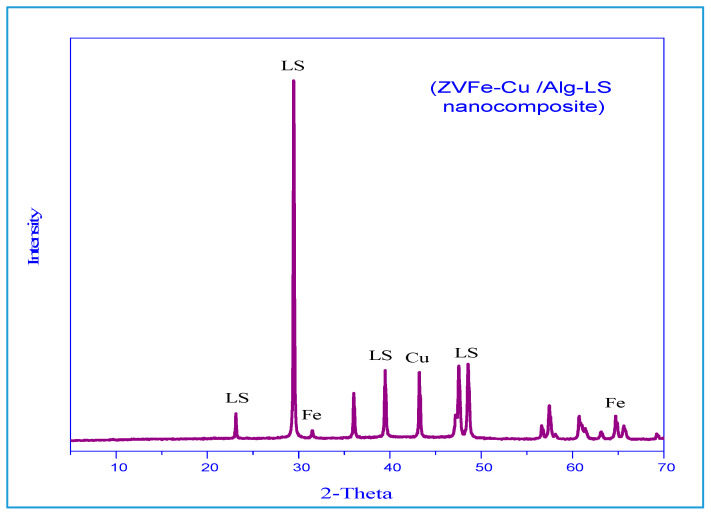
XRD of the Fe–Cu/Alg–LS nanocomposite.

Spectra were analyzed for the phase purity of the Fe–Cu/Alg–LS nanocomposite as shown in Figure 2. The characteristic diffraction peaks located at 43.7° (111) indicated formation of Cu nanocrystals [33]. The diffraction peak observed at 2θ = 44.77° are indexed to (101) denoted the crystalline phase for Fe nanoparticles [34]. The peaks at 29.4 and 47.1° indicated the presence of calcite [35]. The results reveal the formation of copper, ferric and calcite that guarantees the good synthesis of the Fe–Cu/Alg–LS nanocomposites.

#### 3.1.3. SEM and EDX Study

**Figure 3:**
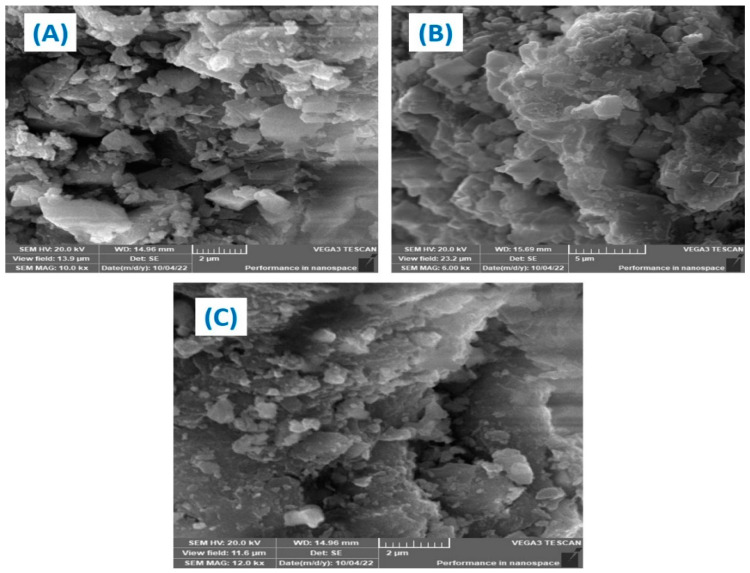
SEM of (**A**) Fe–Cu/Alg–LS nanocomposite, (**B**) Fe–Cu/Alg–LS—loaded CIP, (**C**) Fe–Cu/Alg–LS—loaded LEV.

Figure 3 shows the SEM analysis of the generated. Fe–Cu/Alg–LS nanocomposites both before and after the adsorption of ciprofloxacin (CIP) and levofloxacin (LEV). Figure 3 provides SEM images (A–D). The micrographs display rough surfaces, many holes, and nanoparticles dispersed throughout the sample. The surface exhibits an uneven surface overall for the adsorption of the designated antibiotics. Figure 3A’s surface morphologies showed more pores than Figure 3B,C, indicating that the nanocomposite has enough space for the adsorption process to take place. According to Figure 3B,C, the nanocomposite had less pores due to the CIP and LEV that cover the composite.

**Figure 4:**
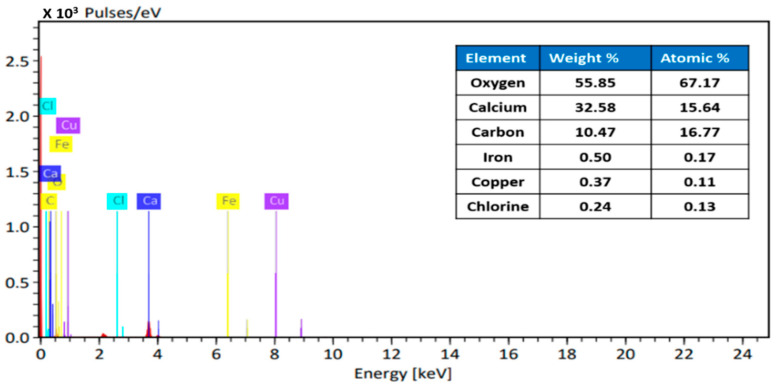
EDX of Fe–Cu/Alg–LS nanocomposite.

The EDX analysis of Fe–Cu/Alg–LS nanocomposites are shown in Figure 4. The EDX analysis of the Fe–Cu/Alg–LS nanocomposites before adsorption reveals the peaks corresponding to oxygen, carbon, cupper, ferric and calcium elements. Thus, EDX guarantees the good synthesis of the Fe–Cu/Alg–LS nanocomposites.

#### 3.1.4. Transmission Electron Microscopy Study

**Figure 5:**
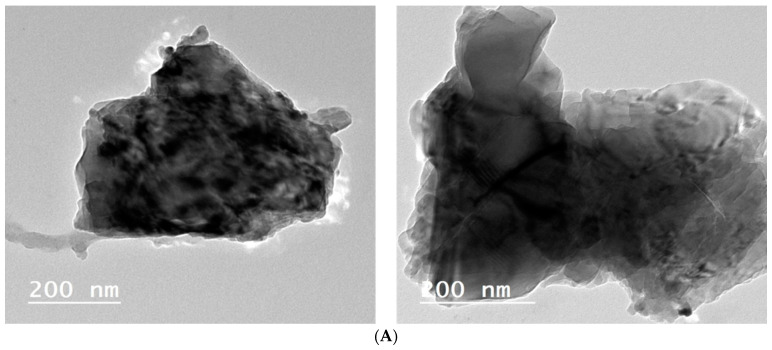

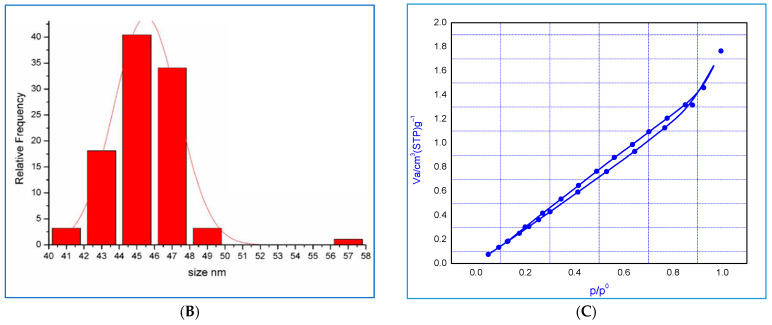
(**A**) TEM analysis of the ZVFe–Cu/Alg–LS nanocomposite (**B**) particle size distribution for ZVFe–Cu/Alg–LS nanocomposite (**C**) Adsorption–desorption nitrogen isotherms.

The TEM and particular area electron diffraction images of the Fe–Cu/Alg–LS nanocomposite is provided in Figure 5. From TEM micrographs, it is clear that the constructed Fe–Cu/Alg–LS nanocomposite exhibited a multilayer structure. This rough surface indicates that nucleation occurred. As seen in Figure 5A, the sample’s microstructure and porosity are well suited for enhanced absorption. The wide range in particle size was shown in a histogram of the particle size distribution generated from the TEM images. The particles have an average diameter of 45.54 nm and range in size from 40 to 50 nm.

#### 3.1.5. BET Adsorption—Desorption Measurements

The surface area and porous structure were examined with N_2_ adsorption–desorption tests. The N_2_ adsorption–desorption isotherms curve of the as-prepared aerogels obtained at 77 K are shown in Figure 5C. Figure 5C reveals a type-IV isotherm for the as-prepared samples calcined at different temperatures, indicating the existence of a mesoporous structure. The surface area data showed that the pore volume and surface area of the Fe–Cu/Alg–LS nanocomposite were 0.04432 cm^3^ g^−1^ and 21.05 m^2^ g^−1^, respectively, as listed in Table 2.

### 3.2. Performance of the Fe–Cu/Alg–LS Nanocomposite

#### 3.2.1. Effect of pH

**Figure 6:**
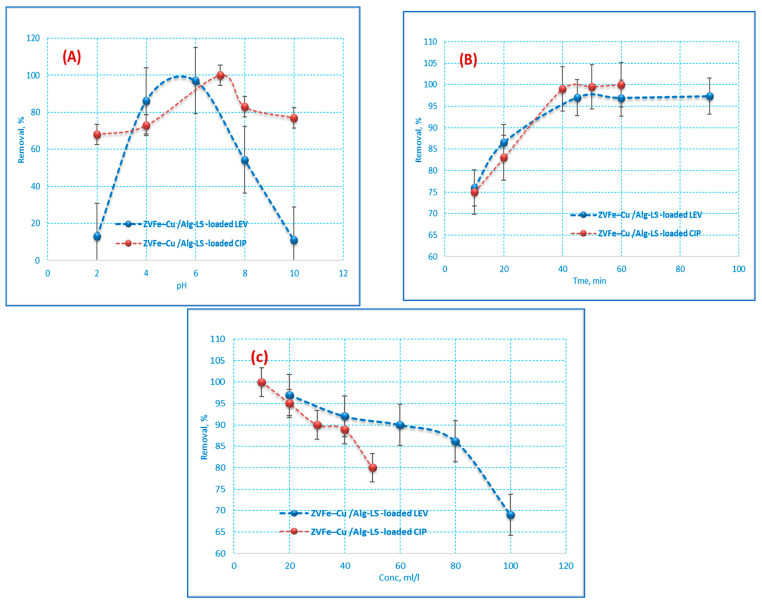
Influences of (**A**) pH, (**B**) contact time and (**C**) initial (CIP and LEV) concentration on the adsorption of CIP and LEV by 0.2 g/25 mL of the nanocomposite at pH 6 (CIP) 7 (LEV) and a contact time of (40 min (CIP) 45 min (LEV)).

An extremely significant factor that affects the removal efficiency of an adsorbent in wastewater treatment is the pH of the solution since the adsorption efficiency is influenced by the pH of the medium. CIP and LEV adsorption was adjusted to make the solution acidic, neutral, and alkaline (2–10). According to Figure 6A, the maximum CIP and LEV removal was obtained at pH 6 and 7, respectively. It is well recognized that the solubility of CIP and LEV is a function of pH, which is explained by the presence of different CIP and LEV chemical species at the different pH values. At low pH values, a highly soluble CIP^+^ and LEV^+^ species occurs and its fraction value decreases as pH values move from 2 to 7, where the pKa constant value (carboxylic acid group) is located [36]. Finally, as the pH value continues to increase to higher than 7, CIP^+^ and LEV^+^ becomes more soluble because of the appearance of the CIP^−^ and LEV- species [37,38]. This behavior can be described through the relationship between CIP and LEV total charge and the surface charge of the Fe–Cu/Alg–LS nanocomposite. As the pH increases up to 6 and 7, the cationic form (CIP^+^ and LEV^+^) is present, the negative Fe–Cu/Alg–LS nanocomposite surface will perform a significant adsorption of the pollutant. Moreover, the high efficiency may be attributed to the increase of adsorbent surface area and greater availability of adsorption active sites. The removal efficiency decreases significantly after the initial pH value reaches 7. This performance can be associated with the presence of the anionic CIP and LEV form (CIP^−^ and LEV^−^), which can produce repulsive interactions with the Fe–Cu/Alg–LS nanocomposite negative surface [39].

#### 3.2.2. Contact Time Effect

Contact time is one of the important influences in the adsorption of the CIP and LEV onto the Fe–Cu/Alg–LS. The effect of contact time on CIP and LEV adsorption on the Fe–Cu/Alg–LS nanocomposite at concentrations of 20 ppm (CIP),10 ppm (LEV) using 0.2 g/25 mL of the nanocomposite and pH 6 (CIP) and 7 (LEV) is presented in Figure 6B. The results illustrate that in CIP and LEV removal efficiency increased to 97.3% and 100% with time. Furthermore, Figure 6B exhibited that the adsorption rate was quick in the first period of time and moderate after 40 min. This may be qualified to the accessibility of abundant free active sites on Fe–Cu/Alg–LS at the initial adsorption stage for CIP and LEV sorption. The rate became very slow after 40 min, and no appreciable CIP and LEV removal was achieved. Hence, equilibrium was reached at about 40 min for CIP and 45 min for LEV. The number of existing active sites reduced with time, and eventually, the adsorbent becomes saturated [40,41]. Consequently, beyond the equilibrium time, no significant uptake of CIP and LEV took place as depicted in Figure 6B. This result could be ascribed to an increase in electrostatic interactions between the surfaces of adsorbents and adsorbates.

#### 3.2.3. Effect of the CIP and LEV Concentrations

Using 0.2 g/25 mL of the nanocomposite, the impact of CIP and LEV concentrations on the adsorption process was investigated at concentrations ranging from 10 to 100 ppm. In addition, the applied pH was (6 for CIP and 7 For LEV), and a contact time of (45 min for CIP and 40 min for LEV), as shown in Figure 6C. As expected, the increase in the concentration of CIP and LEV has a negative effect on the removal efficiency. Moreover, at high concentrations of antibiotics, the adsorbent surface was saturated with pollutants which decreased the adsorption uptake. Consequently, the removal efficiency was found to be decreased from 97% to 69% for CIP and from 100% to 80% for LEV.

### 3.3. Kinetic Models

The pseudo-first-order, pseudo-second-order, and intraparticle diffusion models were engaged to evaluate the adsorption kinetics of the Fe–Cu/Alg–LS nanocomposite. [37]. The optimum situations were conventional as pH 6 for (CIP) 7 for (LEV), Fe–Cu/Alg–LS G nanocomposite mass of 0.2 g/25 mL, a contact time of (40 min (CIP), 45 min (LEV)), and 20 ppm (CIP), 10 ppm (LEV) as the initial concentrations.

#### 3.3.1. Pseudo-First-Order Reaction Kinetics

**Figure 7:**
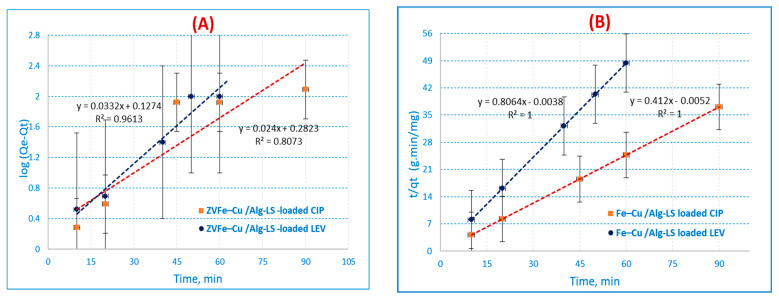

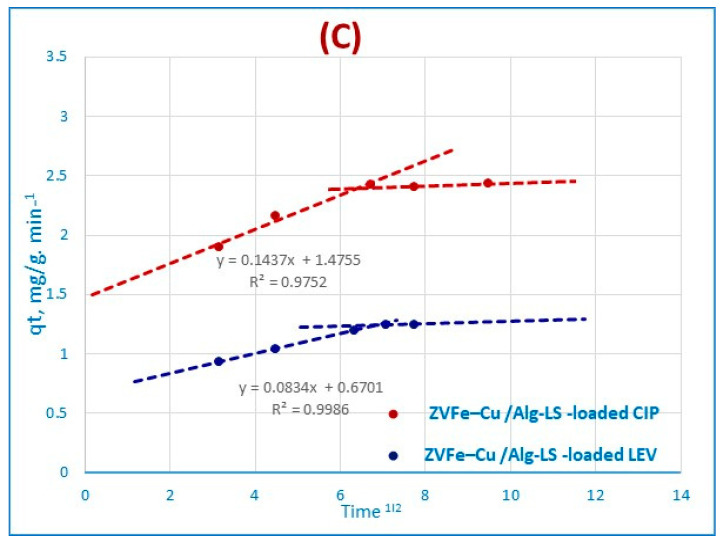
Adsorption kinetics: (**A**) pseudo-first-order reaction (PFORE), (**B**) pseudo-second-order reaction (PSORE), (**C**) Morris–Weber equation for CIP and LEV on the Fe–Cu/Alg–LS nanocomposite (sorption time, (40 min (CIP) 45 min. (LEV) by 0.2 g/25 mL of the nanocomposite at pH 6 (CIP) and 7 (LEV).

Figure 7A [42] provides a description of the PFOR reaction kinetics equation. The following equation is used to represent the current starting phase:

Log (q_e_ − q_t_) − log q_e_ = − K_ads_ t/2.303 (3)

where q_t_ (mg/g) represents the adsorption capacity at time t. K_ads_ (min^−1^) stands for the rate constant of PFOR adsorption.

In this study, a linear relationship was recognized for the adsorption of CIP and LEV ions onto the Fe–Cu/Alg–LS nanocomposite. The values of q_e_ and k_ads_ were measured from the slope and intercept by plotting log (q_e_−q_t_) versus t. The PFOR kinetics are illustrated in Figure 7A. The outcomes exhibited correlation coefficients (R^2^ = 0.8073, 0.9613) for CIP and LEV. The collected data show that the pseudo-first-order model has a poor fit for the adsorption of CIP and LEV onto the Fe–Cu/Alg–LS nanocomposite.

#### 3.3.2. Pseudo-Second-Order Reaction

The PSOR kinetic model [43] is illustrated in the following equation:

t/q = 1/K_2_q_e2_ + t/q_e_ (4)

The PSOR rate constant, denoted by K_2_ (g/mg/min), is shown in Figure 7B. When t/qt is plotted versus t, the slopes and intercepts determine the values of the rate constant K_2_, equilibrium adsorption capacity q_e_, and correlation coefficient (R^2^).

PSOR correlation coefficients (R^2^) for the Fe–Cu/Alg–LS nanocomposite in Table 3 maintained high values. The outcomes exhibited high correlation coefficients (R^2^ = 1) for both CIP and LEV. The statistics imply that the CIP and LEV adsorption suitable for the pseudo-second-order kinetics.

#### 3.3.3. Morris–Weber Kinetic Equation

The Morris–Weber Equation (5) [44] can be used to represent the intraparticle mass transfer diffusion, as shown in Figure 7C.

q = K_d_ (t)^1/2^ (5)

where the uptake of metal ions is denoted by the symbol q (g/g), the intraparticle mass transfer diffusion rate constant is denoted by K_d_, and the square root of time is denoted by the symbol t^1/2^. Only in the shorter stage, if the intraparticle diffusion and adsorption data overlapped, would it occur. The first part is linear, which is related to the boundary layer effect, as shown by the Morris–Weber equation in Figure 7C. However, the intraparticle diffusion effect may be related to the second component [45]. The fact that practically all sorption occurs within the first 40 min for CIP and 45 min for LEV, with a clear linear trend, indicates that the porosity of nanocomposites exceeds the resistance influencing intraparticle diffusion [46]. For CIP and LEV adsorption, the intraparticle diffusion rate constant value K_d_ was estimated to be 0.0831 and 0.0727 (g/g min^−1^), respectively, onto the Fe–Cu/Alg–LG nanocomposite, suggesting CIP and LEV ions move to the composite. The values of K_d_ for both antibiotics represent the rate of diffusion of pollutants towards the pore of nanocomposite, accordingly the rate of diffusion of CIP is higher than LEV onto nanocomposite. The kinetic modeling with the PFOR, PSOR and Morris–Weber equations are detailed in Table 3.

### 3.4. Isotherm Model

To adequately understand the adsorption process, isotherm studies are required [47]. The Langmuir, Freundlich, and Dubinin–Radushkevich isotherm models were used to study the adsorption process. The Fe–Cu/Alg–LS nanocomposite was 0.2 g/25 mL in mass, with contact times of 40 min for CIP (20 ppm) and 45 min for LEV (10 ppm) according to the optimized experimental conditions.

**Figure 8:**
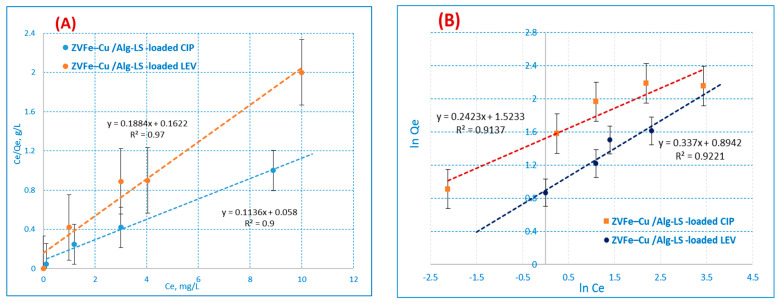
(**A**) Langmuir (**B**) Freundlich adsorption isotherm.

The Langmuir isotherm was used to explain the adsorption of any substance on a homogeneous surface with minimal interaction between the molecules that had been adsorbed [48]. The model assumes a homogeneous uptake in accordance with the saturation level of the monolayer on the surface with the highest adsorption. The following gives an illustration of the Langmuir linear equation model [49]:

Ce/q_e_ = 1/K_L_ q_max_ + (1/q_max_)·C_e_ (6)

where K_L_ (L·mg^−1^) denotes the monolayer’s maximum adsorption capacity and q_max_ (mg.g^−1^) denotes its maximum capacity for sorption heat. Figure 8A,B illustrate the Langmuir adsorption isotherm that was constructed on the basis of monolayer adsorption through the adsorption process. The equilibrium absorption of the homogeneous surface of the adsorbents is explained by the Langmuir model.

The Freundlich model, particularly for heterogeneous surfaces [50,51], is one of the first empirical equations compatible with the exponential distribution of active centers as follows:

ln q_e_ = ln K_f_ + 1/*n* ln C_e_ (7)

If K_f_ denotes adsorption capacity, n denotes intensity, and K_f_ is a crucial and relative indicator of adsorption capacity; it denotes a beneficial adsorption extent. Adsorption is considered suitable when n is greater than 1 [52]. The results demonstrate that the Langmuir model performed better than the Freundlich model in describing the experimental data of the Fe–Cu/Alg–LS nanocomposites. The values for the correlation coefficient (R^2^) are described in Table 3. For both CIP and LEV adsorption, the R^2^ values from the Langmuir model data were 0.9731 and 0.9990, above those of the Freundlich isotherm. The displacement of CIP and LEV ions appears to be a monolayer covered on the surface of the Fe–Cu/Alg–LS nanocomposite, according to the adsorption results. As a result, the outcomes closely matched the Langmuir model.

#### Dubinin–Radushkevich Isotherm

This model fits exceedingly well with the Gaussian energy distribution and adsorption techniques that were used on a heterogeneous surface. The D-R equation is as follows [53]:

ln q = ln q_(D-R)_ − ßε^2^ (8)

ε = RT ln(1 + 1/C_e_) (9)

When the ideal gas constant, R, is taken into account, the theoretical adsorption capacity, q(D-R) (mg·g^−1^), the activity coefficient, ß (mol^2^ kJ^−2^), the Polanyi potential(ε), T (absolute temperature in K), and E (kJ mol^−1^), represented as the free energy change, are as follows:

E = 1/(2ß)^1/2^ (10)

The E value can be used to identify the type of reaction. Physical forces are predicted to have an impact on the adsorption process if E < 8 kJ mol^−1^. If E is between 8 and 16 kJ mol^−1^, chemical ion exchange takes place during the sorption process. Particle diffusion may also be used to determine the sorption process if E is more than 16 kJ mol^−1^ [54]. Table 4 provides a list of the D-R model simulation data. E values for the absorption of CIP and LEV ions onto the Fe–Cu/Alg–LS nanocomposite were 0.7624 and 0.7446 kJ mol^−1^. As a result, if E is less than 8 kJ mol^−1^, physical adsorption will affect the sorption [55].

### 3.5. Sorption Thermodynamics

To estimate the thermodynamic action of the CIP and LEV adsorbed onto the Fe–Cu/Alg–LS nanocomposite, the thermodynamic factors were assessed in order to determine the thermodynamic viability and spontaneous nature of the process of adsorption. At varied temperatures (30, 40, and 60 °C), the findings were recorded. The formulae listed below were used to calculate the thermodynamic factors [56,57]:

∆G° = −RT ln K_d_ (11)

∆G° = ∆H° − T∆S° (12)

ln K_d_ = −∆H°/RT + ∆S°/R (13)

where T is the absolute temperature (K), K_d_ is the distribution coefficient, and R is the gas constant (8.314 J mol^−1^ K^−1^). Using Equation (11), the Gibbs free energy was calculated. Furthermore, using Equation (12), G° might be calculated from H. Using Equation (13), the thermodynamic variables S° and H° were calculated (from the intercept and slope). The data showed that the amount of CIP and LEV ions taken up by nanocomposites slightly decreased in direct proportion to the temperature increase. In contrast to the adsorbent particles, the rise in degree of temperature increased the pollutants’ solubility in a bulk solution to a larger extent [58]. Table 5 illustrates the Fe–Cu/Alg–LS nanocomposite components’ thermodynamic sorption response to CIP and LEV ions.

Negative G° show that the adsorption process is feasible and spontaneous. Additionally, negative findings of H° also suggest that LEV was adsorbed onto the Fe–Cu/Alg–LS nanocomposite in an exothermic manner. Positive readings of H° show that CIP was adsorbed endothermically. Given how CIP and LEV adsorbed onto the surfaces of the adsorbents, negative S° for the Fe–Cu/Alg–LS adsorbent demonstrated that randomness declined at the solid–liquid interfaces, demonstrating that the adsorption was energetically stable [59]. For G° values under 80 kJ mol^−1^, the sorption was of a physical origin. However, if G° was between 80 and 400 kJ mol^−1^, it might have been chemical [60]. Table 4 shows the “G” values, which show that CIP and LEV sorption were of a physical origin. These results support the D-R isotherm.

### 3.6. A Comparison Study

A comparison study between the sorption capacities for CIP and LEV with other sorbents in literature are listed in Table 6.

## 4. Conclusions

Through the use of FTIR, SEM, EDX, and TEM, it was demonstrated that the Fe–Cu/Alg–LS nanocomposite was successfully designed and used to remove the CIP and LEV ions from aquatic solutions. The elimination of CIP and LEV ions was successfully accomplished by the Fe–Cu/Alg–LS nanocomposite. It was also fairly stable at high temperatures. The pharmaceutical concentration and pH level of the solution have a significant impact on sorption capacity. The ideal pH values of 6 and 7 for the adsorption of CIP and LEV ions from contaminated solutions, respectively, were carefully selected. The kinetic models of CIP and LEV ions onto the Fe–Cu/Alg–LS nanocomposite were fitted using the Langmuir adsorption and pseudo-second-order rate equation. After calculating the thermodynamic variables, it was determined that the reaction was spontaneous, exothermic for LEV and endothermic for CIP. The Fe–Cu/Alg–LS nanocomposite sorption was physical. The developed composites demonstrated the potential to be used as an adsorbent in water treatment.

## Figures and Tables

**Table 1:** Physicochemical properties of ciprofloxacin and levofloxacin.

	Ciprofloxacin (CIP) [22]	Levofloxacin (LEV)
Structure	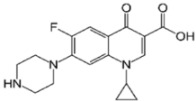	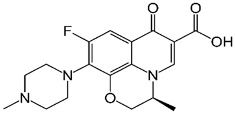
Molecular formula	C_17_H_18_FN_3_O_3_	C_18_H_20_FN_3_O_4_
Usage	Antibiotic	Antibiotic
Molecular weight (g mol^−1^)	331.346	361.373
pKa	6.09	5.59 (acid) and 7.94 (base) [23]
Water solubility (g L^−1^) at 20 °C	36	1.44 [24]

**Table 2:** The pore size distribution adsorption results (surface area, pore volume and average pore radius).

Sample	Surface Area (m^2^ g^−1^)	Pore Volume (cm^3^ g^−1^)	Pore Radius (nm)
Fe–Cu/Alg–LS	21.0506	0.04432	1.92176

**Table 3:** Kinetic modeling with the PFOR, PSOR and Morris–Weber equations.

Kinetic Models	Parameter	Ciprofloxacin (CIP)	Levofloxacin (LEV)
	q_e_, exp (mg g^−1^)	2.425	1.24
PFOR	q_e_, cal (mg g^−1^)	1.915	1.34
	K_ads_ (min^−1^)	0.4120	0.8064
	R^2^	0.8073	0.9613
PSOR	q_e_, cal (mg g^−1^)	2.427	1.24
	K_2_ (g mg^−1^ min^−1^)	0.00007	1.157
	R^2^	1	1
Morris–Weber	K_d_ (mg g^−1^ min^0.5^)	0.0831	0.0727
	R^2^	0.1884	0.9783

**Table 4:** Sorption isotherms.

Kinetic isotherm	Parameter	Ciprofloxacin (CIP)	Levofloxacin (LEV)
Langmuir	q_e_, cal. (mg g^−1^)	8.84	5.3
	K_L_ (L mg^−1^)	0.0012	0.0011
	R^2^	0.9731	0.9990
Freundlich	K_F_ (mol*^n^*^−1^ L*^n^* g^−1^)	4.5850	2.7150
	*n*	4.12	3.60
	R^2^	0.9137	0.9221
D–R model	E (kJ mol^−1^)	0.7624	0.7446
	q_(D-R)_ (mg g^−1^)	8.4350	4.4260
	R^2^	0.9402	0.8088

**Table 5:** Thermodynamic conditions using 0.2 g/25 mL of the nanocomposite at pH 6 for CIP (10 ppm) and 7 for LEV (20 ppm), with contact times of 40 min and 45 min for CIP and LEV, respectively.

Parameter	T (K)	A%	ln K_L_	∆H° (KJ·mol^−1^)	∆S° (J·mol^−1^·K^−1^)	∆G° (kJ·mol^−1^)	R^2^
Ciprofloxacin (CIP)	303	97.0	1.25	10.75	−46.12	−3.148	0.9621
	313	97.2	1.46			−3.812	
	333	97.6	1.65			−4.568	
levofloxacin (LEV)	303	99.0	2.50	−12.39	−20.49	−6.26	0.8929
	313	98.6	2.20			−5.72	
	333	98.4	2.03			−5.45	

**Table 6:** A comparison between the sorption capacities for CIP and LEV with other sorbents in previous work.

Ciprofloxacin			
The Sorbent	Adsorption Capacity, mg/g	Conditions	References
Fe–Cu Bimetallic Supported on Alginate–Limestone Nanocomposite	8.8	20 ppm, 45 min.	Current research
Chemically prepared carbon from date palm leaflets	44.6	Ci = 200 ppm, 2880 min.	[9]
Pillared Clays	122.1	Ci = 18–500 ppm, 1440 min.	[61]
A chemically modified bamboo biochar was prepared from bamboo sawdust	78.43	Ci = 20 ppm, 46 min.	[42]
Protein-modified nanosilica (ProMNS)	85	Ci = 10 ppm, 90 min.	[62]
Activated carbon from Mangosteen Peel	29.76	Ci = 300 ppm, 60 min.	[63]
Fe clay cellulose-acrylamide beads	57.84	Ci= 0.01, ppm	[3]
**Levofloxacin**			
Fe–Cu Bimetallic Supported on Alginate–Limestone Nanocomposite	8.8	10 ppm, 40 min.	Current research
Clay nanotubes	442	Ci =10 ppm,1800 min.	[64]
Fe clay cellulose-acrylamide beads	38.01	Ci= 0.01 ppm	[3]
Magnetite (Fe_3_O_4_—gINPs) nanoparticles from Moringa olifera	22.47	Ci= 4 ppm, 1440 min.	[65]
Hydroxyapatite nanopowder	157.09 (uncalcined Nanohydroxyapatite)	Ci= 25 ppm, 80 min.	[66]

## Data Availability

Data on the compounds are available from the authors.

## Sample Availability

Samples of the compounds are available from the authors.
